# Effects of meteorological factors and groundwater depths on sap flow density of *Populus euphratica* in a desert oasis, Taklamakan Desert, China

**DOI:** 10.3389/fpls.2024.1330426

**Published:** 2024-02-09

**Authors:** Yanbo Wan, Lei Peng, Abudureyimu Anwaier, Haobo Shi, Dinghao Li, Yu Ma, Qingdong Shi

**Affiliations:** ^1^ College of Ecology and Environment, Xinjiang University, Urumqi, China; ^2^ Key Laboratory of Oasis Ecology, Xinjiang University, Urumqi, China; ^3^ Urumqi Comprehensive Survey Center on Natural Resources, China Geological Survey, Urumqi, China

**Keywords:** sap flow, solar radiation, vapor pressure deficit, groundwater, *Populus euphratica*

## Abstract

Accurate estimation of desert vegetation transpiration is key to regulating desert water resources of desert ecosystems. Sap flow density (SFD) can indirectly reflect a tree’s transpiration consumption, and it has been affected by climate warming and groundwater depths in desert ecosystems. Sap flow responses to meteorological conditions and groundwater depths are further affected by tree of different sizes. However, how meteorological factors and groundwater depths affects tree sap flow among tree sizes remains poorly understand. In this study, a 50 × 50 m *P. euphratica* stand was selected as a sample plot in the hinterland of the Taklamakan Desert, and the SFD of *P. euphratica* of different sizes was measured continuously using the thermal diffusion technique from May to October of 2021 and 2022. The results showed that SFD of large *P. euphratica* was consistently higher than that of small *P. euphratica* in 2021 and 2022. and the SFD of *P. euphratica* was significantly and positively correlated with solar radiation (Rad) and vapor pressure deficit (VPD), and the correlation was higher than that of the air temperature (Ta) and relative humidity (RH), and also showed a strong non-linear relationship. Analysis of the hour-by-hour relationship between *P. euphratica* SFD and VPD and Rad showed a strong hysteresis. Throughout the growing season, there was no significant relationship between SFD of *P. euphratica* and groundwater depth, VPD and Rad were still the main controlling factors of SFD in different groundwater depths. However, during the period of relative groundwater deficit, the effect of groundwater depth on the SFD of *P. euphratica* increased, and the small *P. euphratica* was more sensitive, indicating that the small *P. euphratica* was more susceptible to groundwater changes. This study emphasized that Rad and VPD were the main drivers of SFD during the growing season, as well as differences in the response of different sizes of *P. euphratica* to groundwater changes. The results of the study provide a scientific basis for future modeling of transpiration consumption in *P. euphratica* forests in desert oases, as well as the regulation and allocation of water resources.

## 1 Introduction

Desert vegetation resources are an important part of the ecosystem in arid desert areas and are of considerable importance in maintaining ecosystem stability and combating desertification (Lang et al., 2015; Aishan et al., 2016). Given the shortage of water resources, the growth and distribution of desert vegetation are severely restricted (Reynolds et al., 1999; Sun et al., 2009; Si et al., 2014). Accurate estimation of desert vegetation transpiration, as an important component of water consumption (Douglas and Sheil, 2018), is key to regulating desert water resources and quantifying the vegetation carrying capacity of desert ecosystems. However, Understanding the environmental mechanism of forest transpiration is a prerequisite for accurate estimation of vegetation transpiration.

Trunk sap flow measurements can indirectly reflect a tree’s transpiration consumption. Trunk sap flow is the process of water loss from the plant body due to leaf transpiration, which causes water to be transported through the xylem to the leaves (Michael et al., 2006; Cohen et al., 2001). At present, the method of determining trunk sap flow is mainly the thermal technique method, which has the advantages of high sensitivity, low damage to trees, continuous field observation, and automated data collection (Siqueira et al., 2020). It can accurately reflect the water transport condition in trees, and the characteristics of plants’ utilization of water, and their response to the environment, and is also an important parameter for verifying and revising the transpiration model and the root system water absorption model (Wullschleger et al., 1998; Wullschleger and Norby, 2001; Gong et al., 2006). Therefore, trunk sap flow has become one of the key indicators for analyzing the water consumption characteristics of trees and studying the water transport mechanism of trees.

Vegetation trunk sap flow has been studied extensively, with the focus on transpiration and canopy conductance (Daley and Phillips, 2006; Jung et al., 2011; Wang et al., 2017a; Wan et al., 2020). Others have studied the relationship between environmental variables and sap flow, showing that solar radiation (Rad), vapor pressure deficit (VPD), air temperature (Ta), relative humidity (RH) and soil moisture are the main factors influencing sap flow (Lagergren and Lindroth, 2002; Chen et al., 2011; Wang et al., 2017b; Wang et al., 2020). However, there were differences in influencing factors for sap flow, mainly depending on the climatic conditions of the environment in which the tree located, the species, and the age of the tree. For the study of vegetation in arid zone, Keyimu et al. (2017) found that Rad, Ta and RH were the main influencing factors for sap flow density (SFD) of *Populus euphratica*. Deng et al. (2011) found that sap flow rates differed significantly between different groundwater gradients of *Toona sinensis*. Meanwhile, when there was sufficient groundwater available, the sap flow rates were predominantly influenced by Rad and VPD. However, in desert areas with extremely single water source and scarce rainfall, groundwater is basically the main water source for vegetation growth. At present, although studies on the relationship between groundwater and sap flow have been carried out in some areas, it is still unclear whether the impact of groundwater decline on sap flow is increased, and how sap flow of different tree sizes responds to the decline in groundwater depth.

Daliyabuyi is a natural pristine oasis formed by the tail of the Kriya River. The oasis is located in the hinterland of the Taklamakan Desert in Northwestern China, which is a region with an arid climate and relatively little rainfall (< 5 mm). *Populus euphratica* (Salicaceae) is the dominant species in this oasis and it plays an important ecological role in maintaining the stability of oasis communities. In recent years, the construction of large reservoirs in the upper reaches of Keria River has seriously affected the distribution of water resources in time, space and volume, and caused serious degradation of oasis vegetation (Shi et al., 2021). Based on the above situation, we used isotope techniques to study the water use characteristics and water use strategies of *P. euphratica* in oasis (Wan et al., 2022), However, to date, there has been a lack of research on the variation in transpiration water consumption and its influencing factors in *P. euphratica*.

In this study, our aim was to clarify the variation of SFD in *P. euphratica* of different sizes and the relationship with meteorological factors and groundwater depth. Based on these research objectives, three hypotheses were proposed, that is, (1) Rad and VPD are the main driving factors affecting the SFD of *P. euphratica*. (2) Changes in groundwater depth always affect changes in SFD of *P. euphratica*. and an increase in groundwater depth will result in a greater effect on the SFD of *P. euphratica*. (3) Small *P. euphratica* was affected by the groundwater depth more than large *P. euphratica*. In order to test these hypotheses, we selected a *P. euphratica* sample plot and continuously monitored the SFD of different sizes of *P. euphratica* in the sample plot using the thermal diffusion technique, while continuously monitoring the meteorological and groundwater depth in the sample plot. The study findings can provide scientific guidance for maintaining the stability of poplar forest communities in arid desert areas and developing rational water management measures.

## 2 Materials and methods

### 2.1 Study area

**Figure 1:**
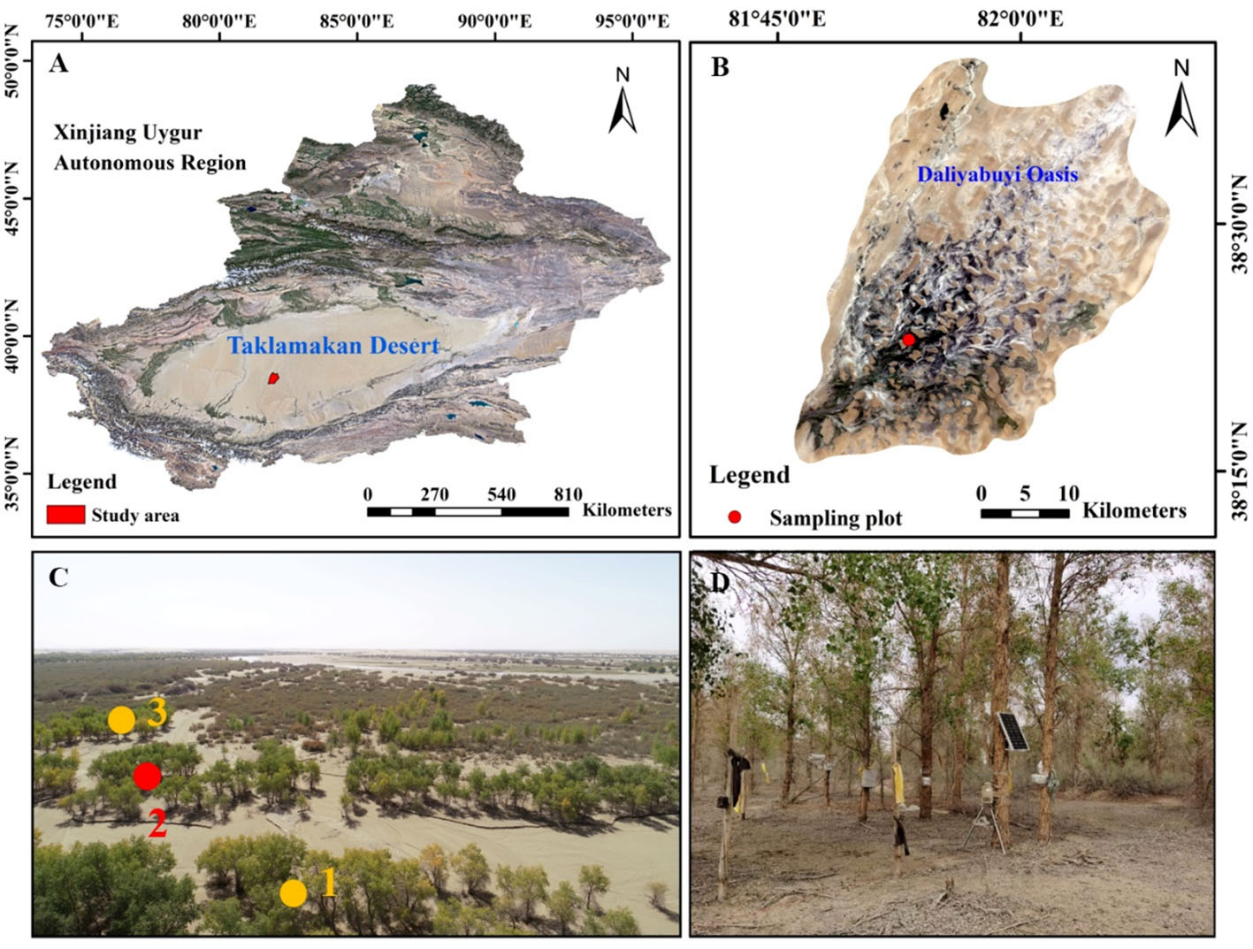
Location maps of the research area (**A**: Xinjiang, **B**: Daliyabuyi Oasis). Photograph of the landscape: yellow dots represent the vegetation survey plots, and the red dot represent the intensive plots **(C)**. The intensive plots for continuous measurement sap flow **(D)**.

The study area is located in Yutian County, Xinjiang Uygur Autonomous Region (38°16’-38°37’ N, 81°05’-81°46’ E, 1100–1300 m above sea level) (**Figure 1**), a desert hinterland oasis formed by the confluence of the Kriya River in the Taklamakan Desert. The core area of the oasis is 324 km^2^. It has a representative warm temperate arid desert climate. The average annual temperature is 12.1°C, the average annual precipitation is 2.53 mm, the average annual potential evaporation is 2480 mm (Wan et al., 2022). The region is in the desert hinterland, with a high number of sandstorm days and severe wind and sand hazards. The oasis vegetation is relatively homogeneous and sparsely distributed, consisting mainly of xerophytic trees, shrubs and annual or perennial herbs, including *Populus euphratica*, *Tamarix chinensis*, *Phragmites communis*, and *Alhagi sparsifolia*. The main soil type is mainly sandy.

### 2.2 Sample plot survey and sample tree selection

In this study, a groundwater level observation well in the middle of the oasis was selected, and three 50 × 50 m *P. euphratica* sample plots were established near the observation well, and a detailed vegetation survey was carried out in 2021. Parameters such as the height, diameter at breast height (DBH), and crown width of each tree in the sample plots were measured by the classical method (**Table 1**), and it was found through the analyses of the data from the sample plot surveys that the *P. euphratica* growths did not differ much under the same moisture gradient, and in order to make the sample plots representative, among the three plots, we selected sample 2, whose basic parameters were at the middle level, as an intensive sample plot, and carried out the long-term and continuous observation of the trunk sap flow.

**Table 1:** The characteristics of three sample plots, Plot 2 was selected as intensive plot.

Sample plot No.	Average DBH (cm)	Average tree height (m)	Average canopy diameter (m)	Number
1	29.15	10.52	4.40	79
2	27.36	10.34	4.36	92
3	26.54	9.75	3.88	85

Study area belongs to a natural desert oasis with sparse distribution of *P. euphratica*. In order to clarify the characteristics and influencing factors of the SFD of *P. euphratica* of different sizes. We classified *P. euphratica* into small (DBH ≤27), and large (DBH >27 cm) trees based on vegetation data of the sample plot. Three representative healthy trees were chosen from each DBH ranges, and six trees in total were monitored for SFD. The basic characteristics of the sample tree are shown in **Table 2**.

**Table 2:** Basic characteristics of the six sample trees of *P. euphratica*.

	Tree No.	DBH (cm)	Tree Height (m)	Canopy diameter (m)	Sapwood thickness (cm)
Small *P. euphratica*	1	14.30	7.32	1.85	2.07
	2	16.40	8.81	3.72	2.29
	3	21.50	10.50	4.20	2.88
Large *P. euphratica*	1	30.80	11.43	4.81	3.89
	2	43.60	11.85	7.45	5.20
	3	48.20	12.15	7.83	5.73

### 2.3 Sap flow density measurements and calculations

SFD in the trunks of six *P. euphratica* trees was measured continuously using thermal diffusion with heat dissipation sensors, with each set of sensor probes (SF-L, Ecomatik, Munich, Germany) consisting of two probes 1.2 mm in diameter and 30 mm long (three additional 10 mm probes were added for small trees in 2022). The equipment was continuously powered by a solar panel connected to a 12 V colloidal battery. To avoid thermal effects caused by direct sunlight, it was mounted at breast height on the north side of the tree trunk and wrapped in aluminum foil to protect it from solar radiation.

The trunk SFD was derived from the temperature difference between the two probes based on the principle established by Granier that the temperature difference between the thermal diffusion probes is closely related to the sap flow density, and calculated according to Equation 1 (Wang et al., 2021):


(1) $$J_{s}=0.714\;{\left(\frac{△T_{M}-△T}{△T}\right)}^{1.231}$$

Where J_s_ is the single trunk SFD (ml/cm^−2^·min^-1^), ΔT is the temperature difference between the heating probe and the sensor probe (°C), and ΔT_M_ is the maximum temperature difference between the two needles during each day (°C).

Data were collected every 30 min using the Logger CR-1000 data logger (Campbell Scientific Co.). Measurements were taken over two growing seasons (MayO-October) in 2021 and 2022.

### 2.4 Leaf water content and carbon isotope measurements

Fully exposed, mature and healthy leaves from the upper part of the canopy were collected from different orientations of the six sample trees, two portions were collected from each sample tree, each portion was about 50 g. One portion was used for the determination of leaf water content (fresh weight was measured in the field), and the other portion was used for leaf carbon isotope measurements, and the leaves were put into handmade paper bags separately and brought back to the laboratory for the determination of dry weight and carbon isotopes of the leaves. Sampling time was July 2021. The method of determination is described in the article (Wan et al., 2022). The carbon isotope calculation formula was as follows (Equation 2):


(2) $$\delta^{13}C=\left(\frac{R_{sample}}{R_{standard}}-1\right)\times 1000‰$$

The isotopic compositions were reported in standard δ-notation that represent ‰ deviations from the Vienna Standard Mean Ocean Water (VSMOW). R_sample_ is the ratio of heavy and light element richness in the sample (^13^Csam/^12^Csam), and R_stadard_ is the ratio of heavy and light element richness of the national universal reference material (^13^C_std_/^12^C_std_).

### 2.5 Meteorological observations

An automatic weather station (Weatherhawk232, Weatherhawk, Logan, UT, USA) was set up in an open location near the test site to continuously collect three meteorological data, that is, solar radiation (Rad, W/m^2^), air temperature (Ta, °C) and relative humidity (RH, %), with data being recorded at five-minute intervals. Measurements were taken over two growing seasons (May-October) in 2021 and 2022. Based on the air temperature and relative humidity, the vapor pressure deficit (VPD, kPa) was calculated according to Equations 3 and 4 (Campbell and Norman, 1998):


(3) $$E=0.611\times e^{\frac{17.502\times Ta}{Ta+240.97}}$$

(4) $$VPD=E-\frac{E\times RH}{100}$$

Where E is the vapor pressure (kPa), Ta is the air temperature (°C), and RH is the relative humidity of the air (%).

### 2.6 Measurement of groundwater depth

Groundwater data were obtained from observation wells near the sample site, in which water level recorders (HOBO U20L-01, ONSET Company, USA) were deployed for long-term monitoring of groundwater depth. The groundwater depth was considered as the depth from the surface to the water surface of the borehole. The instrument was protected by a PVC cap and data was recorded and stored every 4 h.

### 2.7 Statistical analysis

Since the sapwood width of small *P. euphratica* trees is smaller than the probe length (30 mm) in 2021 (**Table 1**), the SFD of small *P. euphratica* trees is overestimated or underestimated. In the growing season of 2022, three additional 10 mm probes will be added to the small trees, the SFD of small trees in 2021 will then be corrected based on the sap density determined by the 10 mm and 30 mm probes in 2022. Dix and Aubrey (2021) found that calibration can improve the accuracy of tree transpiration estimates based on SF. However, in this study, due to the small sample size of trees, the data could not be effectively calibrated when estimating transpiration. Therefore, this study focused on the relationship of SFD to meteorology and groundwater depth, rather than the study of the exact absolute values of SF, and the resulting errors do not have an impact on the results of the study.

In this study, when we conducted the analysis of SFD with environmental factors, the data for May–June were missing because of a power supply problem in the early days of the weather station in 2021. Therefore, we used the SFD and meteorological factors for July–October, with the rest of the data from May–October, to create averaged dataset that were used to analyze SFD with meteorological factors and groundwater depth, respectively. We used pearson correlation analysis to analyze the SFD and environmental factors to filter out the main influencing factors. Different models, that is, linear, S-type, and Gompertz, were tested to fit the data and we only retained the model based on the highest R^2^ value. Rad used the S-type (3 - parameter) equation and VPD used the Gompertz (3 - parameter) equations. Normalized data, that is, hourly observations divided by the daily maxima, were used to demonstrate the time lag between hourly SFD and Rad and VPD during the growing season.

**Figure 2:**
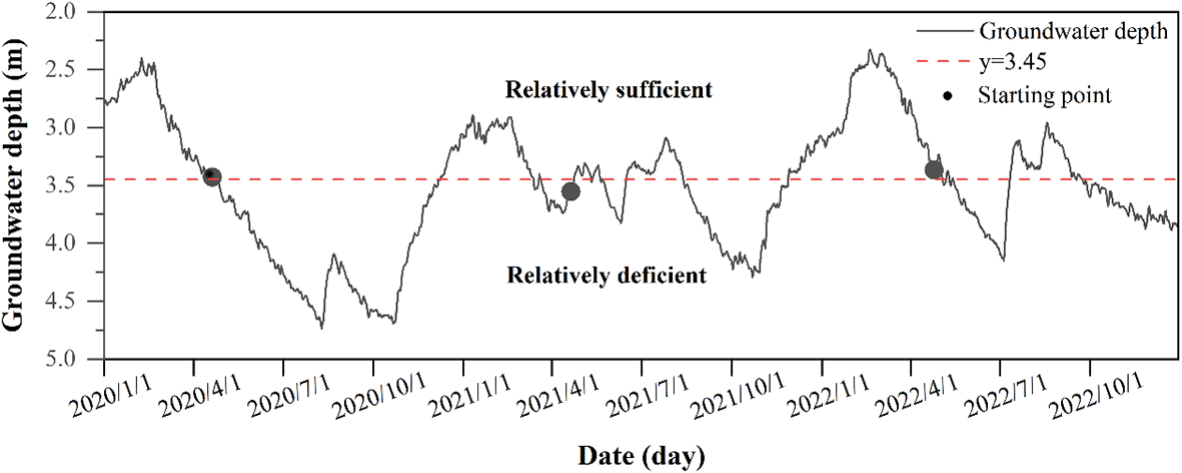
Variations in groundwater depth from 2020 to 2022. The starting point represents the annual germination time of *P. euphratica*. y=3.45 represents the average groundwater depth at the beginning of the growing season of *P. euphratica* in three years.

Through years of monitoring by phenological camera, we found that the time point of *P. euphratica* germination is basically around 20 April every year, so we take 20 April as the beginning of the growing season. At the same time, through the monitoring of groundwater depth in the observation wells for three years, it was found that at the beginning of the growing season, the groundwater depth level was basically maintained at the same level, and we took the average value of the groundwater depth at the beginning of the growing season for three years as the basic point of groundwater depth level, and divided the groundwater depth into two periods, which were the period of relative sufficiency (≤ 3.45 m) and relative insufficiency (> 3.45 m), to explore the effect of the groundwater depth on the SFD of *P. euphratica* (**Figure 2**).

## 3 Results

### 3.1 Variation in meteorological factors and groundwater depth

**Figure 3:**
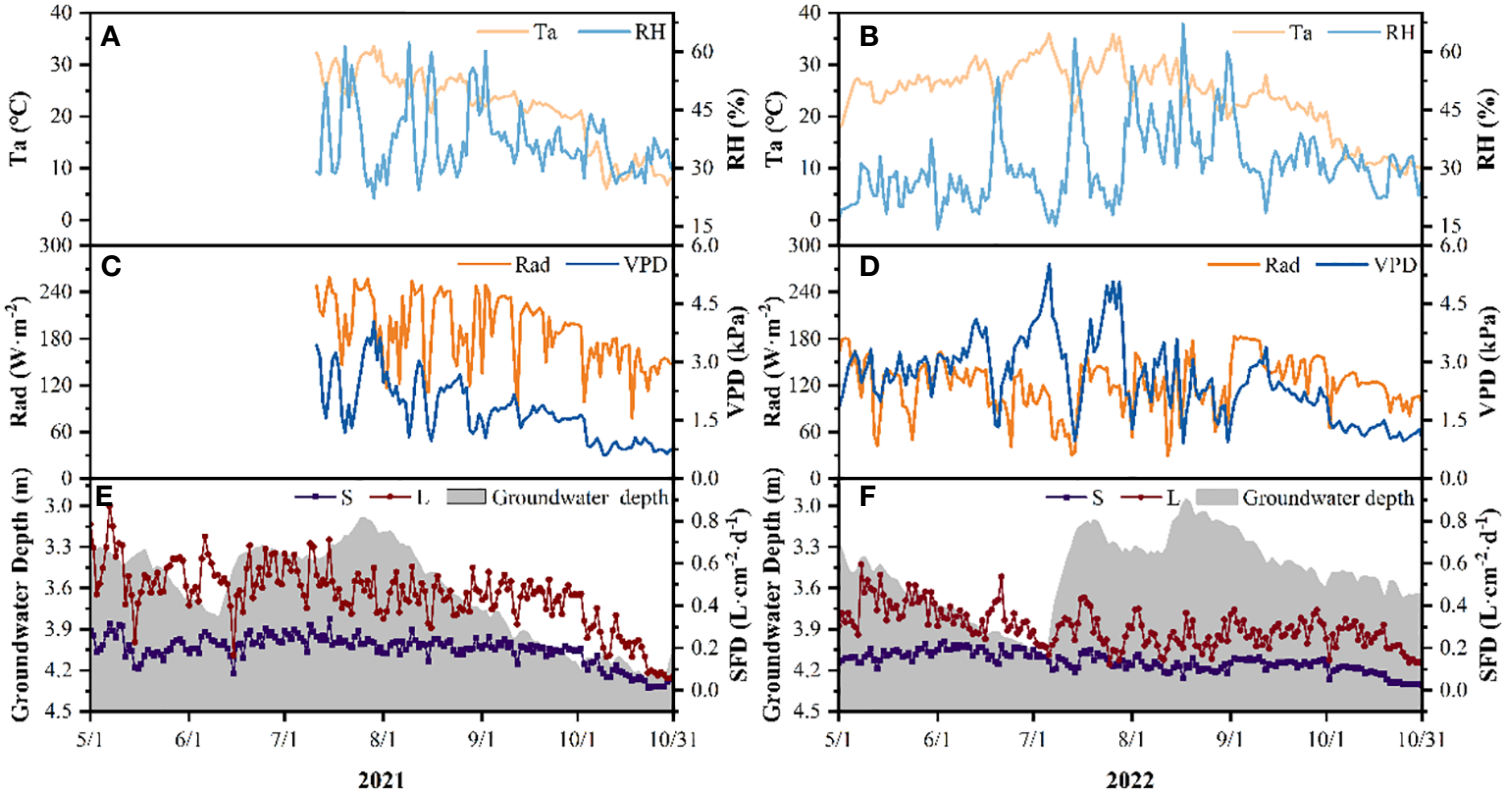
Characteristics of meteorological factors (Ta, air temperature; RH, relative humidity; Rad, solar radiation; VPD, vapor pressure deficit), groundwater depths and sap flow density (SFD) of *P. euphratica* (S, small; L, large) for the growing seasons (May-October) in 2021 and 2022 in the study area (**A**, **C**, **E**: TA and RH, RAD and VPD, SFD and groundwater depth change characteristics in 2021; **B**, **D**, **F**: TA and RH, RAD and VPD, SFD and groundwater depth change characteristics in 2022). meteorological data from May-June 2021 are missing.

**Figure 3** shows the characteristics of the meteorological factors and groundwater depth for the growing seasons (May-October) in 2021 and 2022 in the study area. In 2021, Ta, RH, Rad and VPD all gradually decreased from July to October. The ranges of Ta and RH were -6.12–33.61°C and 22.33–62.40%, respectively, with mean values of 21.41°C and 36.85% (**Figure 3A**). Meanwhile, the ranges of Rad and VPD were 78.34–259.06 W·m^−2^ and 0.61–4.04 kPa, respectively, with mean values of 189.82 W·m^−2^ and 1.75 kPa, respectively (**Figure 3C**). In 2022, Ta, RH and VPD showed a trend of first increasing (May-June) and then decreasing (July-October), while the change of RAD was different and did not show a significant increase (**Figures 3B, D**). From May to October, the ranges of Ta, RH, Rad and VPD were 8.87–35.94°C, 14.36–67.13%, 29.50–183.36 W·m^−2^ and 0.92–5.53 kPa, respectively, with mean values of 24.00°C, 30.89%, 121.25 W·m^−2^ and 2.55 kPa. Compared to 2021, Rad in 2022 decreased by 68.57 W·m^−2^, VPD in 2022 increased by 0.80 kPa.

The groundwater depth differed obviously between the two study years (**Figures 3E, F**). In 2021, the groundwater depth showed a trend of increasing first and then decreasing, ranging from 3.09–4.29 m, the maximum and minimum values appeared on 22 October and 25 July, respectively, with an average value of 3.65m. In 2022, the groundwater depth showed a trend of decreasing first, then increasing, and finally decreasing, ranging from 2.96–4.15 m. The maximum and minimum values appeared on 18 August and 5 July, respectively, with an average value of 3.50 m. The groundwater depth in 2022 was 0.15 m higher than that in 2021.

### 3.2 Variation in sap flow density

**Figure 4:**
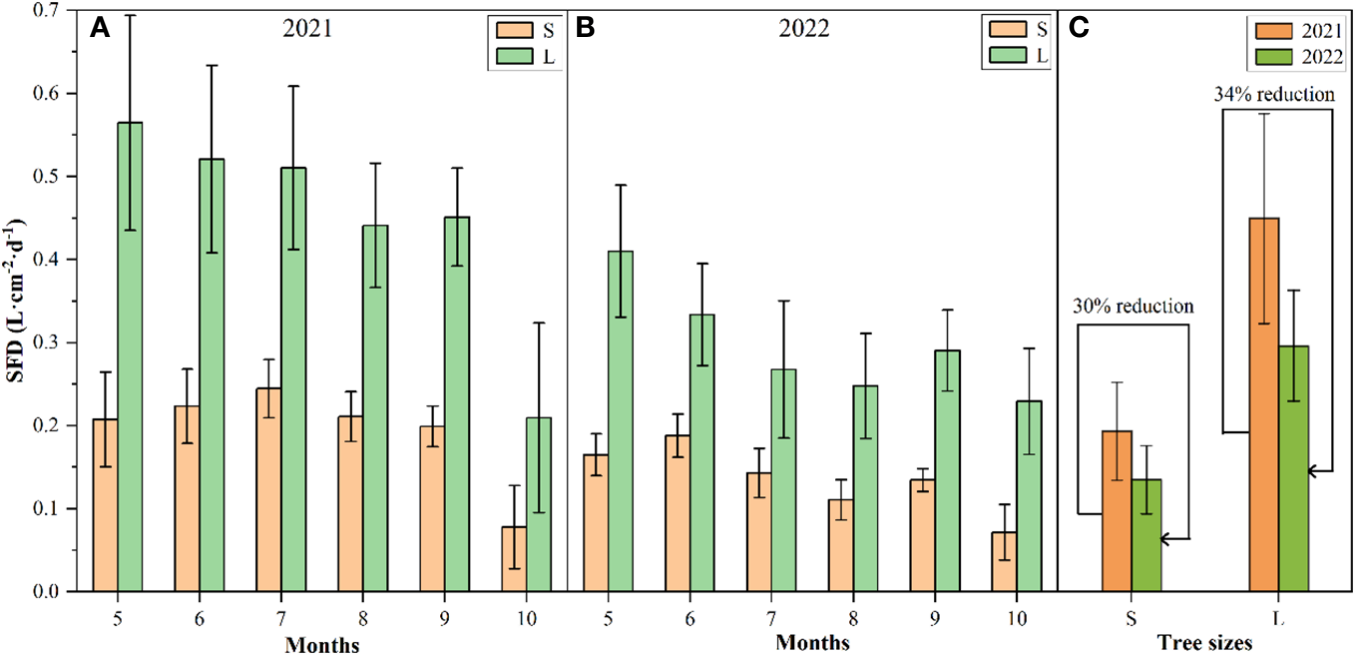
Sap flow density (SFD) characteristics of P. euphratica (S, small; L, large) during the growing season in 2021 and 2022 (**A** and **B**: monthly size change, **C**: size change throughout the growing season). The error bar represents the mean value and standard deviation.

There had different seasonal trend of the sap flow density (SFD) of P. *euphratica* between different sizes in the growing seasons of 2021 and 2022, that is, the SFD of large *P. euphratica* showed a trend of first increasing and then decreasing, and that of small *P. euphratica* showed a decreasing trend. The SFD of large *P. euphratica* was always higher than that of small *P. euphratica* (**Figures 3E, F**). Among them, the maximum SFD of large *P. euphratica* all appeared in May, with an average value of 0.56L·cm^-2^·d^-1^ and 0.41L·cm^-2^·d^-1^ in 2021 and 2022, respectively. while the maximum SFD of small *P. euphratica* had a slight difference between the two growing seasons, the maximum SFD appeared in July in 2021, with an average value of 0.22 L·cm^-2^·d^-1^. In 2022, it appeared in June, with an average of 0.19 L·cm^-2^·d^-1^ (**Figures 4A, B**).

For the growing season (May to October), the mean values of SFD of the large and small *P. euphratica* was 0.45 L·cm^-2^·d^-1^ and 0.19 L·cm^-2^·d^-1^ in 2021, respectively, and were 0.30 L·cm^-2^·d^-1^ and 0.14 L·cm^-2^·d^-1^ in 2022, respectively. Compared to 2021, the SFD of large and small *P. euphratica* decreased in 2022, with a 30% decrease in large *P. euphratica*, and a relatively low 34% decrease in small *P. euphratica* (**Figure 4C**).

### 3.3 Impact of meteorological factors on sap flow density

**Figure 5:**
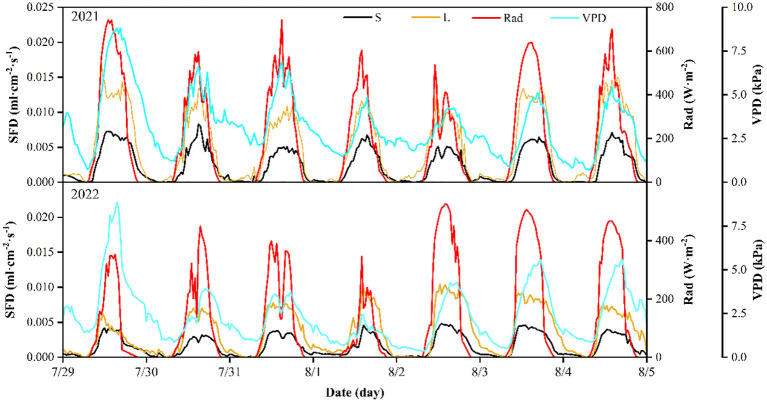
An example of sap flow density (SFD) for *P. euphratica* (S, small; L, large) together with solar radiation (Rad) and vapor pressure deficit (VPD), for one week (peak transpiration period) in 2021 and 2022. All data are half-hour averages.

**Figure 5** shows the average daily SFD of *P. euphratica* trees during representative week of the 2021 and 2022 growing season. Overall, the SFD profiles of the different sizes of *P. euphratica* trees were relatively similar and all showed a bell-shaped curve. The variation in SFD began to rise from near zero after sunrise (8h30), reached a maximum at around 15h30 and then gradually declined to near zero (22h00) until midnight.

The daily variation in SFD follows the trend of VPD and Rad. A comparison of the magnitude of SFD during one week of 2021. It can be found that VPD and Rad were relatively higher on 29 July, 3 August and 4 August, the days with higher SFD, and low on the opposite, including on 2 August (**Figure 5**). Meanwhile, it was found that the time points of daily peak occurrence of different sizes of *P. euphratica* SFD were basically synchronized, and the daily SFD was consistent with the peak of daily Rad most of time. However, there was a lag between the daily SFD and the daily VPD peak. In 2022, the SFD showed the same pattern with Rad and VPD.

**Figure 6:**
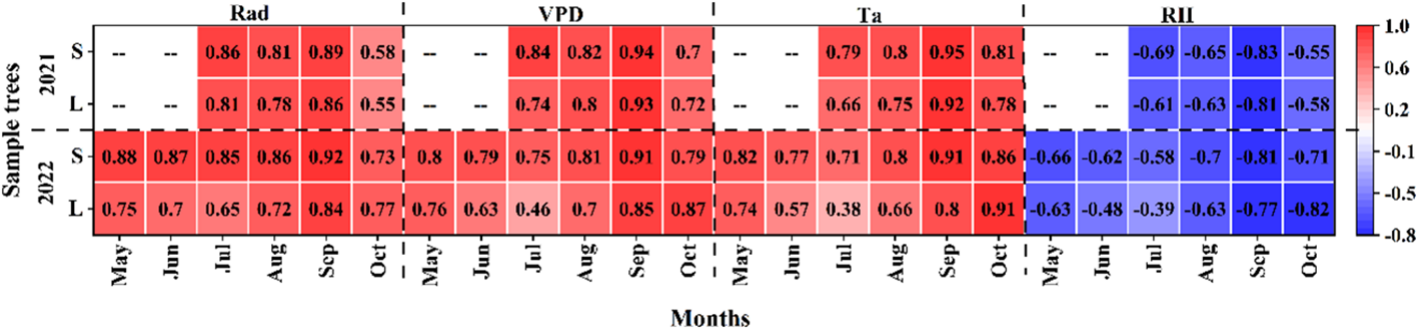
Coefficient of determination (R^2^) between daily sap flow density and each meteorological factor (Ta, air temperature; RH, relative humidity; Rad, solar radiation; VPD, vapor pressure deficit) of *P. euphratica* (S, small; L, large) during the growing season. All meteorological factors were significantly correlated with sap flow density at different time points, that is, they were not marked significantly in the figure.

Daily sap density was significantly positively correlated with VPD, Rad and Ta, and significantly negatively correlated with RH (*P<0.001*) during the growing season in 2021 and 2022 (**Figure 6**). The correlation between small *P. euphratica* and meteorological factors was higher than that of large *P. euphratica*, such as in 2021, the correlations of SFD with VPD, Rad, Ta, and RH of small *P. euphratica* were 0.86, 0.84, 0.79, and -0.69, respectively, while the corresponding values of large *P. euphratica* were 0.81, 0.74, 0.66 and 0.61, respectively, and the same results were seen in 2022. At the same time, we found that the correlation coefficients between SFD and Rad, VPD were higher than Ta and RH, and the VPD was calculated from Ta and RH, and its magnitude was determined by both, that is, the most important meteorological factors affecting SFD in *P. euphratica* were Rad and VPD.

**Figure 7:**
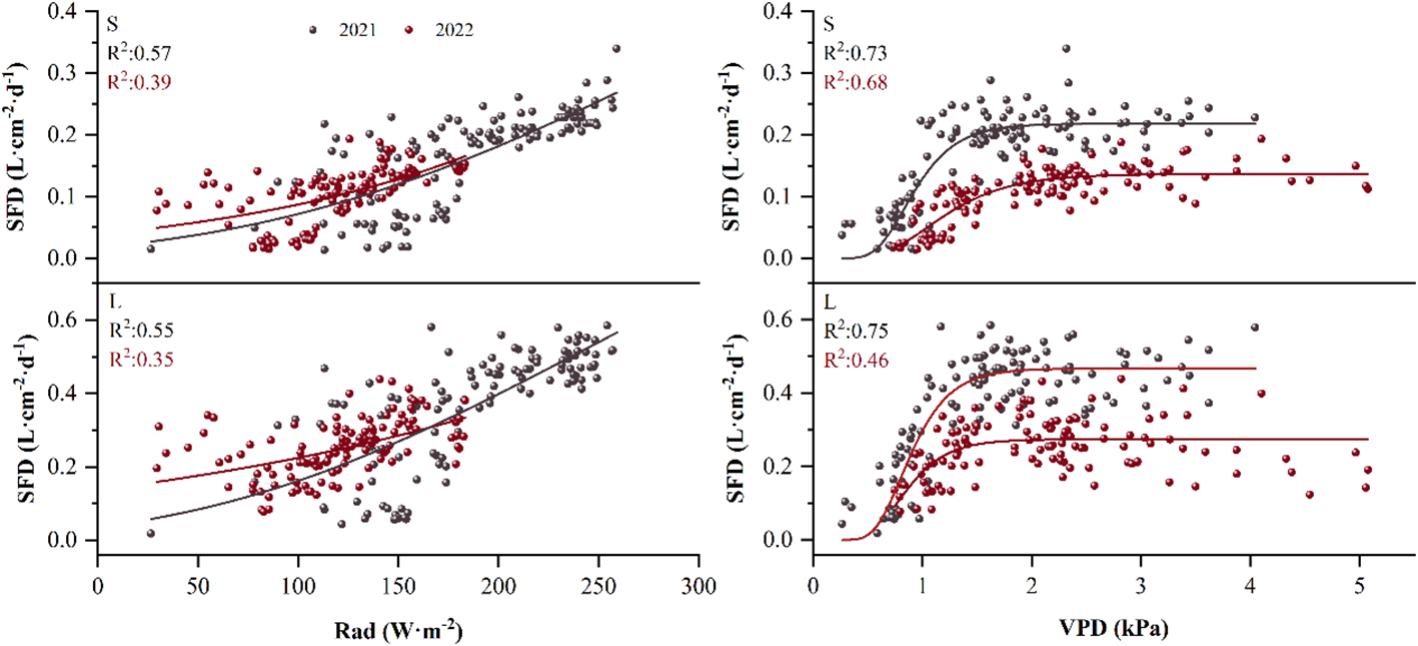
Example of the relationship between daily sap flow density (SFD) and solar radiation (Rad) for *P. euphratica* (S, small; L, large) using the sigmoidal equation for the growing season in 2021 and 2022. Relationship between daily sap flow density and vapor pressure deficit (VPD) for *P. euphratica* using the gompertz equation for the growing season in 2021 and 2022.

**Figure 8:**
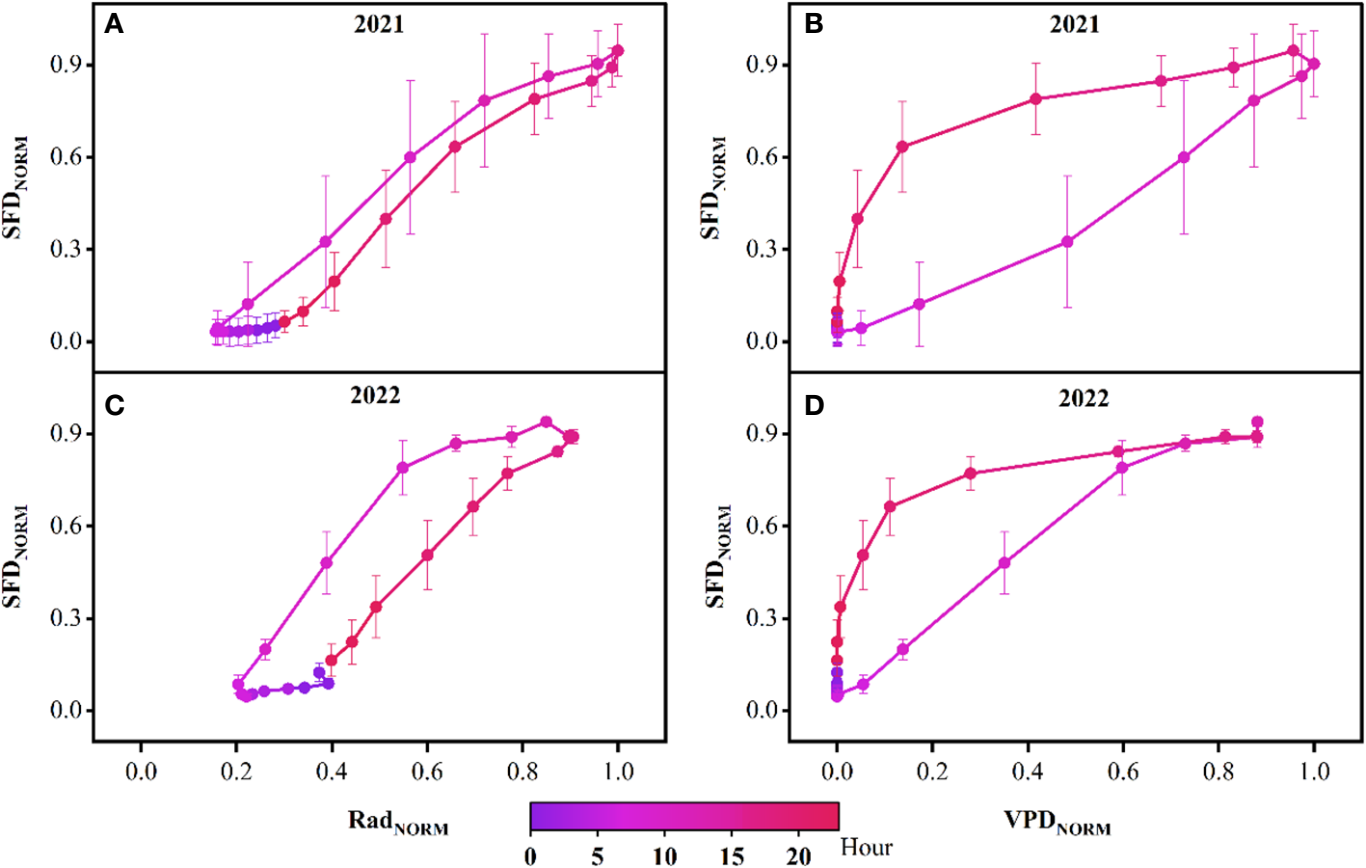
Typical hysteresis loop of normalized sap flow density (SFD_NORM_) data with normalized solar radiation (Rad) and vapor pressure deficit (VPD) on an hourly basis for the growing season in 2021 **(A, B)** and 2022 **(C, D)**. Error bars are mean ± standard deviation based on the six probes.

By curve fitting the daily SFD with Rad and VPD, we found that the sigmoidal equation could most effectively describe the relationship between the daily SFD and Rad, with a fit coefficient R^2^ for small *P. euphratica* of 0.56 and 0.39 in 2021 and 2022, respectively, large *P. euphratica* of 0.55 and 0.35 in 2021 and 2022, respectively. The Gompertz equation could most effectively describe the strong non-linear relationship between the daily SFD and VPD, with a fit coefficient R^2^ for small *P. euphratica* of 0.73 and 0.68 in 2021 and 2022, respectively, large *P. euphratica* of 0.75 and 0.46 in 2021 and 2022, respectively. In general, the fitting effect of small *P. euphratica* was higher than that of large *P. euphratica* (**Figure 7**). Strong diel hysteresis (counterclockwise) was observed for *P. euphratica* for the relationship SF- VPD. The hysteresis was clockwise for Rad, but not as strong as for Rad (**Figure 8**).

### 3.4 Impact of groundwater depth on sap flow density

**Figure 9:**
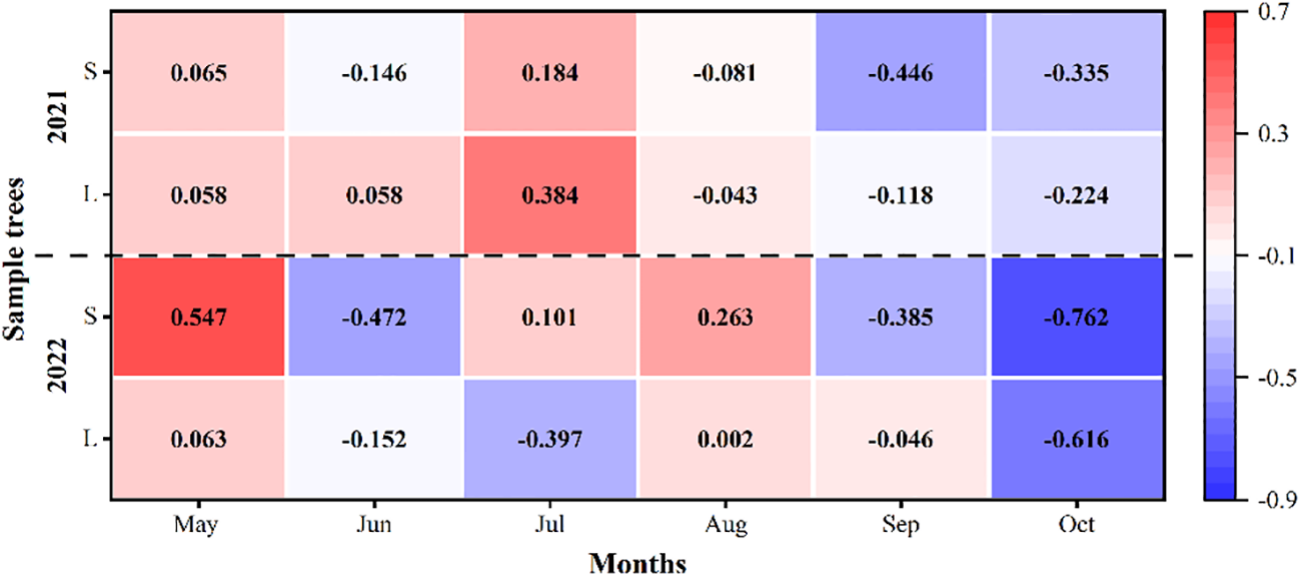
Coefficient of determination (R^2^) between daily sap flow density of *P. euphratica* (S, small; L, large) and groundwater depth during the growing season (May to October) in 2021 and 2022.

There had obvious seasonal differences for the relationship between the SFD of *P. euphratica* and the groundwater depth. In the first and middle of the growing season (May to August), the two were basically positively correlated, while in the late growing season (September to October), they were basically negatively correlated (**Figure 9**). For the whole growing season, the relationship between the SFD of *P. euphratica* and the groundwater depth was not significant. the correlation coefficients between the SFD and the groundwater depth were 0.02 and -0.12 in 2021, respectively, and they were -0.19 and 0.01 in 2022, respectively, for large and small *P. euphratica*.

To further explore the effect of groundwater depth on the SFD of *P. euphratica*, we divided the groundwater depth into two periods, and found that in the period of relatively sufficient moisture conditions, the main factors affecting the SFD were Rad and VPD, and the groundwater depth had almost no effect; whereas in the period of relatively deficient moisture conditions, the correlation coefficient of Rad decreased slightly, and the correlation coefficients of VPD and the groundwater depth increased significantly. It was also found that during periods of relatively deficient moisture conditions, the relationship between small *P. euphratica* and groundwater depth was higher than that of large *P. euphratica* (**Table 2**).

## 4 Discussion

### 4.1 Main meteorological factors affecting sap flow density

In forest ecosystems, tree transpiration is an important component of the water balance of the forest floor and affects the stability of the forest structure (Raz-Yaseef et al., 2012; Ungar et al., 2013). Plant biology, meteorological factors, and the ability of soil water and groundwater to supply water are the main factors controlling plant sap flow (Poyatos et al., 2007; Deng et al., 2011; Pinto et al., 2015; Yan et al., 2021). Meteorological factors control transient changes in plant sap flow and plant biological structure determines sap flow potential. The water supply capacity of soil water and groundwater determines total sap flow (Pinto et al., 2015), and meteorological factors such as Rad, VPD, Ta, and RH are the main factors influencing transient changes in plant sap flow. The sap flow rate of trees has been previously found to be highly significantly and positively correlated with Rad, VPD and Ta, and highly significantly and negatively correlated with RH (Tu et al., 2013; Wu et al., 2017). In addition, sap flow rate can also be influenced by plant species, age, and growth health status (Forster, 2014; Fang et al., 2018).

**Figure 10:**
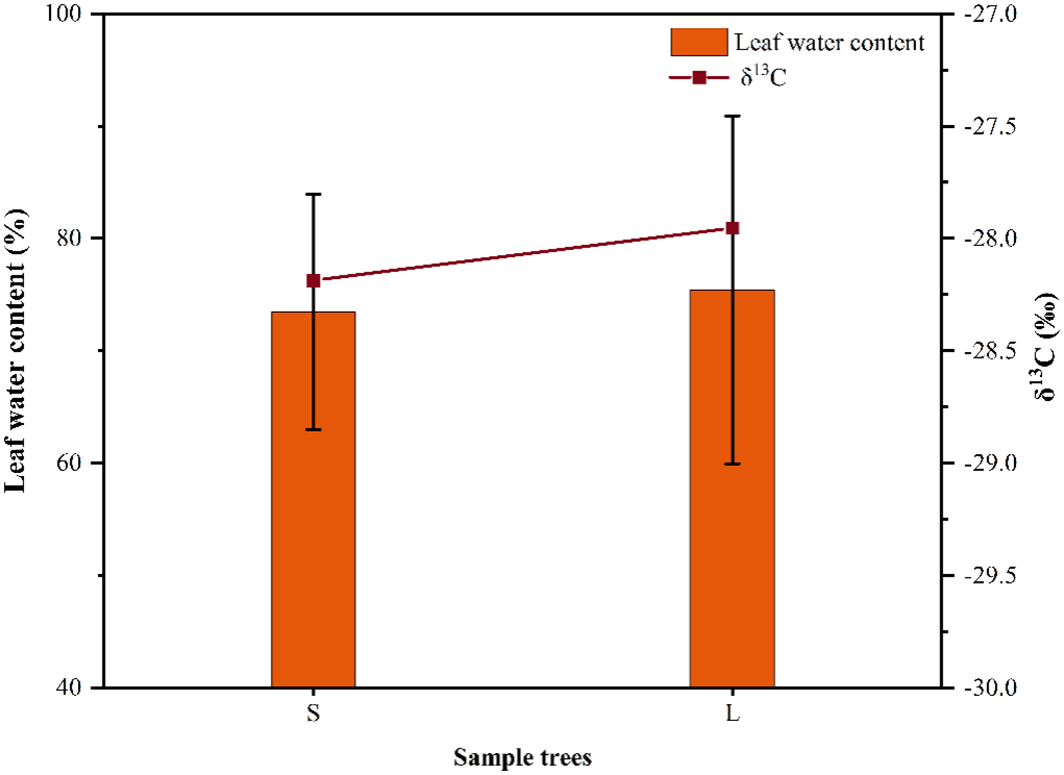
Variation in leaf δ^13^C values and leaf water content of *P. euphratica* (S, small; L, large) in 2021.

In this study, there were significant differences in SFD among different sizes of *P. euphratica* trees, with larger *P. euphratica* having significantly higher SFD than smaller *P. euphratica*. This was predominantly determined by the plant structure, because larger *P. euphratica* have larger canopies and require more water to maintain their growth, which is consistent with the findings of other studies (Drake et al., 2015; Yan et al., 2016). our study found that the *P. euphratica* SFD was significantly smaller in 2022 than in 2021, which was mainly due to the change of environmental factors, the Rad was smaller in 2022 than in 2021, which led to the decrease of the SFD of *P. euphratica*. Meanwhile, we found that the decrease of large (34%) was larger than that of small (30%) *P. euphratica* in 2022, which might be related to the water content and the intrinsic water consumption mechanism of *P. euphratica* leaves. Plant leaf water use efficiency can reveal the intrinsic water consumption mechanism of plants, and carbon isotopes, as the current standard method to study plant water use efficiency, have an irreplaceable role in the study of plant response to environmental changes (Baruch, 2011; Bhusal et al., 2020). Based on the leaf water content and carbon isotopes of different sizes of *P. euphratica* in our sample plots in 2021, and found that the leaf water content and carbon isotopes of large *P. euphratica* were higher than that of small *P. euphratica*, this indicated that the water content and water use efficiency of large *P. euphratica* were relatively high. And thus, when the environmental conditions changed, it had a greater effect on the large *P. euphratica* than on the small *P. euphratica* (**Figure 10**). Meteorological factors control the temporal variation in the sap flow rates of trees. For example, Fernandez et al. (2009) found that VPD was the main environmental factor for the variation of SFD in different tree species; Zheng et al. (2014) found that solar radiation was significantly and positively correlated with the sap flow rate of pike in the arid zone, with the largest correlation coefficient. In this study, Rad, VPD, and Ta showed highly significant positive correlations with SFD. Meanwhile, RH showed highly significant negative correlations with SFD. Among them, the correlation coefficients of Rad and VPD with SFD were higher than those of Ta and RH, that is, Rad and VPD were the most important factors influencing of flow density, which is consistent with the findings of other studies (Tu et al., 2013; Wu et al., 2017), also consistent with our hypothesis (1). Meanwhile, in our study, the *P. euphratica* SFD had a strong nonlinear relationship with the VPD. However, to date, there have been a substantial number of reports on this nonlinear relationship (Ford et al., 2004; Van Herk et al., 2011; Liu et al., 2015). Similarly, Rad has also shown a strong nonlinear relationship for sap flow (Wullschleger and Norby, 2001; Chang et al., 2014; Wang et al., 2017b). Meanwhile, in our study, the nonlinear relationship between SFD and VPD in *P. euphratica* was consistently stronger than that of Rad.

In this study, the daily peaks of SFD were consistent across different sizes of *P. euphratica*, and all lagged Rad and advanced ahead of VPD. In contrast, for the study of peak sap flow in trees of different DBH, height and age, Guo et al. (2019) found that the sap flow of Japanese willows with larger DBH peaked later than those with smaller DBH, and shortened the length of the time lag with the VPD; Gotsch et al. (2018) found that the taller the tree, the greater the water stress, the relatively earlier the stomatal regulation time, and the earlier the time of peak sap flow, leading to an increase in the time lag between peak sap flow and the VPD. Tsuruta et al. (2018) found that the peak sap flow of 43-year-old *Chamaecyparis obtuse* was ahead of that of 23-year-old *Chamaecyparis obtuse*, and both were ahead of the VPD. However, some studies have also found that the effects of tree height, DBH and crown width on the time lag of sap flow are not significant (Yan et al., 2016; Yao et al., 2018; Zhang et al., 2019). The results of our study were the same as the latter and opposite to the former, which was mainly due to the fact that our study area was located in the center of the desert, the density of *P. euphratica* stands was low, both large and small *P. euphratica* could be fully exposed to sunlight, and the moisture conditions of the sample site were sufficient, and the effects of BHD and tree height on the time lag were negligible. We investigated the time-by-time variation of SFD with Rad and VPD in *P. euphratica* and found that the normalized curve of SFD and VPD in *P. euphratica* exhibited a strong time-lag effect (clockwise). Meanwhile, there was a less prominent relationship with Rad (counterclockwise), which is consistent with the results of Li et al. (2016) for *P. euphratica*, Wang et al. (2017a) for *Populus cathayana*, and Xu and Yu. (2020) for *Haloxylon ammodendron*. However, a substantial number of studies have been conducted on time lag effects for different species in different ecosystems (Zeppel et al., 2004; Zheng et al., 2014; Pappas et al., 2018). For the causes of time lag, different researchers have attempted to investigate the causes of time lag in terms of nocturnal transpiration (Yu et al., 2017), delayed transport distance between trunk and leaves (Cermák et al., 2007), and tree water storage (Zhou et al., 2012). In the arid zone, Han et al. (2018) studied the phenomenon of sap flow time lag in vegetation trunks of arid sand areas and found that the relationship between sap flow time lag and nighttime water replenishment was not significant. The correlation with daytime water consumption was highly significant. Meanwhile, our study area was located in a desert area with high temperature and little rainfall. Here, vegetation transpiration water consumption is relatively high, canopy transpiration exceeds vegetation water absorption capacity, and root water absorption replaces water loss in tree tissues, which then results in a time lag effect.

### 4.2 Effect of groundwater depth on sap flow density

In arid desert areas, groundwater is an important source of water for vegetation growth (Zhao et al., 2017; Wu et al., 2018). previous studies on groundwater depth and plant sap flow concluded that the shallower the water table, the greater the plant sap flow (Zhang et al., 2017). In this study, there were seasonal differences in the relationship between *P. euphratica* SFD and groundwater depth, a result that differs from our hypothesis (2). This may be due to the fact that the sample site is located in the upper reaches of the river, which is recharged by the river water, the groundwater depth fluctuates greatly, and the moisture conditions are relatively good in some seasons, so the relationship with the groundwater depth shows seasonal differences. At the same time, we found that the effect of groundwater depth on *P. euphratica* SFD increased when moisture conditions were relatively insufficient, a result consistent with our hypothesis (2). However, the effect of meteorological factors was always much greater than that of groundwater depth. Some studies have confirmed that the groundwater depth less than 3 m is the optimal condition for the growth and development of *P. euphratica*, and 3 ~ 5 m is the better depth for *P. euphratica* growth (Zhao et al., 2012). The average groundwater depths of the two growing seasons in the sample plots of our study were 3.65 and 3.50 m, respectively, with the lowest groundwater depth of 4.29 m, it was much less than 5 m. Therefore, in this study, the relationship between *P. euphratica* SFD and groundwater depth was not significant and was mainly controlled by meteorological factors.

In addition, we found that the effect of groundwater depth on the SFD of small *P. euphratica* was higher than that of large *P. euphratica* when the moisture conditions were relatively deficient (**Table 3**). Previous studies have found that trees of different sizes have different responses to drought (Pretzsch et al., 2018; Stovall et al., 2019; Lopez et al., 2021). For example, Stovall et al. (2019) studied 2 million trees in California and found that large trees were more sensitive to drought. Lopez et al. (2021) studied European forests and found that pine sap flow was more sensitive in large trees during drought, while the exact opposite was true in spruce. The above studies were carried out in temperate forests. However, in arid desert areas, where precipitation is scarce, groundwater is the main water source for vegetation survival, and the distribution of roots determines the ability of vegetation to cope with drought. At the root level, the size of trees is related to the distribution of roots, and the root biomass of large trees is higher than that of small ones (Persson, 1983; Canadell et al., 1996), therefore, the response of small trees to groundwater was more sensitive, which was consistent with our hypothesis (3).

**Table 3:** Correlation analysis of sap flow density of *P. euphratica* (S, small; L, large) and environmental factors (Ta, air temperature; RH, relative humidity; Rad, solar radiation; VPD, vapor pressure deficit) under different water conditions (Pearson correlation).

Year	Sizes	Rad	VPD	Groundwater Depth	Rad	VPD	Groundwater Depth
		Relatively sufficient			Relatively deficient		
2021	S	0.908**	0.815**	0.047	0.799**	0.897**	-0.361**
	L	0.799**	0.694**	0.054	0.759**	0.863**	-0.204**
2022	S	0.876**	0.752**	0.153*	0.805**	0.907**	-0.254**
	L	0.735**	0.543**	0.066	0.836**	0.899**	-0.147*

*P< 0.05.

**P< 0.01.

### 4.3 Deficiencies and prospects

The oasis is located in the center of the Taklamakan Desert, and the *P. euphratica*, as the most important species of this oasis, can block the flow of sand and dust and reduce the frequency of sandstorm disasters in cities on the edge of the desert. Therefore, it is very important to maintain the healthy growth of oasis vegetation. While the groundwater that maintains the survival of oasis *P. euphratica* mainly comes from surface water, the completion of the upstream reservoir in 2018 led to a great restriction on the amount of groundwater recharge in the oasis. At this time, it is particularly important to estimate the water demand of oasis vegetation. In this study, we determined the SFD of *P. euphratica* of different sizes, which can provide basic data support for the calculation of *P. euphratica* water demand, and at the same time, we carried out a study on the relationship between SFD and environmental factors, so as to provide a basis for the establishment and optimization of the water demand model of the whole oasis. In this study, it was found that the relationship between the SFD of *P. euphratica* and meteorological factors was significant, while the relationship with the groundwater depth was weak. However, in environments with insufficient water conditions, is it possible that *P. euphratica* sap flow is mainly affected by groundwater depth? It has been shown that in flooded habitats, *P. euphratica* uses higher water for transpiration, less water for synthesizing organic matter, and low water use efficiency (Liu et al., 2021). Meanwhile, due to the small sample size in this study, the accuracy of the sap flow calculation for large and small trees may be biased. Therefore, we need to set up *P. euphratica* sample plots with different groundwater depths in the later period, monitor more *P. euphratica* trees, and further explore the relationship between *P. euphratica* sap flow and groundwater, so as to achieve accurate estimation of sap flow of *P. euphratica*. Eventually, based on our multi-site monitoring, we can determine the groundwater level with higher water utilization efficiency of *P. euphratica* under the premise of ensuring the healthy growth of *P. euphratica*, so as to make a reasonable plan for the regulation of water resources by the upstream reservoir and the allocation of water resources in the oasis.

## 5 Conclusion

In this study, the relationship between the SFD of *P. euphratica* of different sizes and environmental factors in arid desert areas was investigated. It was concluded that the SFD of *P. euphratica* was controlled by Rad and VPD, and the SFD showed a significant nonlinear relationship with Rad and VPD. We also found a strong hysteresis between the SFD and VPD, while the hysteresis with Rad was less pronounced. Throughout the growing season, the relationship between SFD and groundwater depth was not significant, whereas it showed a negative correlation with SFD when groundwater depth increased, in which the small *P. euphratica* showed more sensitivity, but the effect on SFD was much smaller than that of Rad and VPD. *P. euphratica* is the main dominant species in the region, it is particularly important to carry out regional-scale water consumption estimation of *P. euphratica* forests and to establish transpiration modeling. This study proves that it is reasonable to establish a model based on meteorological factors to estimate the water demand of *P. euphratica* forest in the area with groundwater depth less than 5 m. However, for the estimation of water consumption in the whole oasis, there are still some limitations in our study, such as the lack of monitoring of the SFD of *P. euphratica* growing in deeper groundwater level. In the future, more studies on the SFD of multiple groundwater level samples should be conducted to further improve the accuracy of water consumption estimation of the whole oasis *P. euphratica* forest.

## Data availability statement

The original contributions presented in the study are included in the article/supplementary material. Further inquiries can be directed to the corresponding author.

## Author contributions

YW: Formal Analysis, Writing – original draft, Writing – review & editing. LP: Conceptualization, Writing – review & editing. AA: Investigation, Writing – review & editing. HS: Investigation, Writing – review & editing. DL: Investigation, Writing – review & editing. YM: Writing – review & editing. QS: Writing – review & editing.
